# Detecting Remote Sequence Homology in Disordered Proteins: Discovery of Conserved Motifs in the N-Termini of *Mononegavirales* phosphoproteins

**DOI:** 10.1371/journal.pone.0031719

**Published:** 2012-03-05

**Authors:** David Karlin, Robert Belshaw

**Affiliations:** Department of Zoology, University of Oxford, Oxford, United Kingdom; University College Dublin, Ireland

## Abstract

*Paramyxovirinae* are a large group of viruses that includes *measles virus* and *parainfluenza viruses*. The viral Phosphoprotein (P) plays a central role in viral replication. It is composed of a highly variable, disordered N-terminus and a conserved C-terminus. A second viral protein alternatively expressed, the V protein, also contains the N-terminus of P, fused to a zinc finger. We suspected that, despite their high variability, the N-termini of P/V might all be homologous; however, using standard approaches, we could previously identify sequence conservation only in some *Paramyxovirinae*. We now compared the N-termini using sensitive sequence similarity search programs, able to detect residual similarities unnoticeable by conventional approaches. We discovered that all *Paramyxovirinae* share a short sequence motif in their first 40 amino acids, which we called *soyuz1*. Despite its short length (11–16aa), several arguments allow us to conclude that *soyuz1* probably evolved by homologous descent, unlike linear motifs. Conservation across such evolutionary distances suggests that *soyuz1* plays a crucial role and experimental data suggest that it binds the viral nucleoprotein to prevent its illegitimate self-assembly. In some *Paramyxovirinae*, the N-terminus of P/V contains a second motif, *soyuz2*, which might play a role in blocking interferon signaling. Finally, we discovered that the P of related *Mononegavirales* contain similarly overlooked motifs in their N-termini, and that their C-termini share a previously unnoticed structural similarity suggesting a common origin. Our results suggest several testable hypotheses regarding the replication of *Mononegavirales* and suggest that disordered regions with little overall sequence similarity, common in viral and eukaryotic proteins, might contain currently overlooked motifs (intermediate in length between linear motifs and disordered domains) that could be detected simply by comparing orthologous proteins.

## Introduction

**Figure 1:**
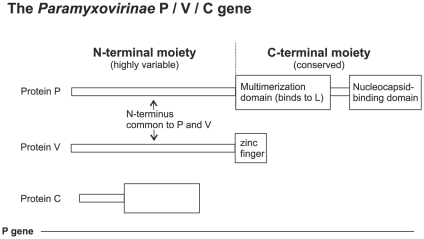
Organization of the *Paramyxovirinae* P gene. The P, V and C proteins are encoded from alternative reading frames. V is produced in all *Paramyxovirinae* genera whereas C is only produced in *henipaviruses*, *morbilliviruses*, and *respiroviruses*.

*Paramyxovirinae* are a large subfamily of viruses containing nine human pathogens such as measles virus, mumps virus and the emergent Hendra and Nipah viruses. The viral Phosphoprotein (P) plays a central role in viral replication and in interferon escape. P plays multiple roles in replication, acting as a co-factor of the viral polymerase (L) and binding to the nucleocapsid [1]. The viral nucleoprotein (N) can self-assemble illegitimately on cellular RNA, and a third function of P is to prevent this by binding N and keeping it in a monomeric form, called N°, until encapsidation occurs [1]. The *Paramyxovirinae* P gene expresses other proteins than P from different reading frames (Figure 1): the protein V, which shares its N-terminus with P but has a different C-terminus (forming a zinc finger), and, in some genera, the protein C, which overlaps the N-terminus of P (Figure 1). All three proteins encoded by the P gene play a role in interferon escape [2]. Experimental studies of P are difficult for many reasons: multiple functions, gene overlaps, abundance of structural disorder in P and N [3], [4], [5], large size of L and the nucleocapsid, and transient interactions.

*Paramyxovirinae* P is composed of two main parts: an N-terminal moiety that is highly variable in sequence and in length, from 150 to 380 amino acids (aa), and is disordered [6], [7], [8], [9], i.e. lacks a defined, stable tertiary structure [10], and a conserved C-terminal moiety comprising a multimerization domain that binds to L, and a nucleocapsid-binding domain (Figure 1). Related viruses from the order *Mononegavirales*, such as *Pneumovirinae*, *Rhabdoviridae* and *Filoviridae*, express a similar protein, usually also called P, which also binds the nucleocapsid, acts as the co-factor of the polymerase, and is also almost always encoded by the second gene of the viral genome. The P of all *Mononegavirales* have a similar organization [11], [12], [13], [14], [15], [16], [17] but there is no apparent sequence or structural similarity in P across all families.

Previously, using standard approaches such as psi-blast [18], we detected sequence similarity in a short region of the N-terminus of some *Paramyxovirinae* P only [5]. However, all *Paramyxovirinae* P are clearly orthologous (i.e. descended from a common ancestor without gene duplication), since their C-termini have statistically significant similarity and they are encoded by genes in the same location [5]. Therefore, we reasoned that their disordered N-terminal moieties might all be also descended from a common ancestor, despite their high variability in sequence and in length. In that case, they might have retained some residual sequence similarity that would have escaped detection by conventional approaches. In order to detect such potential regions, we used sensitive bioinformatics approaches that can detect weak similarities between protein regions: profile-profile comparison and multiple sequence alignment coupled with software that can indicate reliably aligned regions. Motifs found by this approach can be validated by examining their prevalence, their location, their function, and by finding them in newly sequenced viruses that were unknown at the time of the analysis.

We discovered that the N-termini of the P of all 45 species of *Paramyxovirinae* share a short sequence motif within their first 40aa, soyuz1. Disordered regions, particularly of viral proteins, are thought to evolve extremely fast and, to our knowledge, this is the first reported example of sequence conservation in a disordered region between such distantly related viruses. We argue that this conservation suggests an important function for soyuz1 and we propose reasons why it might constitute a good drug target. A second motif, soyuz2, is found downstream of soyuz1 in some *Paramyxovirinae*, and may play a role in blocking the interferon pathway.

We analyzed other *Mononegavirales* P and found that their disordered N-termini also contained conserved motifs of similar length, although these might not be homologous to soyuz1. In addition, their C-termini, despite having different folds, contained a structurally and functionally similar region, suggesting that they might have a common origin.

## Materials and Methods

Our hypothesis is that the disordered N-termini of the phosphoproteins might contain regions that are similar in sequence. The similarity is expected to be weak since it has escaped detection so far. At present, the most sensitive method to detect sequence similarities between two query proteins is to gather homologs of each, to derive two multiple sequence alignments (MSAs), each composed of one query protein and of its homologs, and to compare the two MSAs using profile-profile comparison [19]. A sequence profile is a representation of a multiple alignment, containing information about which amino acids are “allowed” at each position of the alignment and about their probability of occurring. Comparing profiles of two multiple alignments is much more powerful than comparing two single sequences, because the profiles contain information about how each sequence can evolve, and can therefore detect weak similarities that remain after both sequences have evolved apart [19].

Our strategy consists of the following steps: 1) collect sequences of orthologous phosphoproteins; 2) extract their N-terminal regions; 3) group them by genus and align them; 4) identify sequence motifs, i.e. regions having detectable, though possibly statistically subsignificant sequence similarity, using profile-profile comparison and multiple sequence alignment; 5) check that their conservation does not result from the presence of an underlying RNA structure; 6) the final step is to validate motifs that have subsignificant similarity. This can be done by a) obtaining *new sequences* from distantly related viruses (if they also have the motif, it is very unlikely to be spurious); b) examining the *prevalence* of the motifs (a motif found in numerous related species is unlikely to have occurred by chance); c) examine the *location* of the motifs (motifs all occurring in exactly the same position are more likely to result from homologous descent than from convergent evolution); and d) examine *functional data* associated with the motifs. This validation step is performed in the Discussion.

### Sequences used in the study

The accession numbers of the sequences of *Paramyxovirinae* P used in this study, as well as the abbreviations of species names are in Table 1. The accession numbers of the P of *Pneumovirinae*, *Filoviridae*, and *Rhabdoviridae* are in Table 2. Unpublished sequences for the *Rhabdoviridae* genus *ephemerovirus* were kindly provided by P.J. Walker. We did not analyse the P of taxa for which too few sequences were available, i.e. *Bornaviridae* and the recent genus *nyavirus* [20]. The N-terminus of P is defined as the part upstream of the multimerization domain (Figure 1).

**Table 1:** Sequences of *Paramyxovirinae* P or V proteins.

Virus species	Abbreviation	Genus	Accession number
*Atlantic salmon paramyxovirus*	Atlantic PMV	*Respirovirus*-like	B1NLR3
*Avian Paramyxovirus 2*	Avian PMV2	*Avulavirus*	B4Y565
*Avian Paramyxovirus 3*	Avian PMV3	*Avulavirus*	B5L5T6
*Avian Paramyxovirus 4*	Avian PMV4	*Avulavirus*	B6UPM7
*Avian Paramyxovirus 5*	Avian PMV5	*Avulavirus*	D3X604
*Avian Paramyxovirus 6*	Avian PMV6	*Avulavirus*	295810683
*Avian Paramyxovirus 7*	Avian PMV7	*Avulavirus*	224979460
*Avian Paramyxovirus 8*	Avian PMV8	*Avulavirus*	C5I0V3
*Avian Paramyxovirus 9*	Avian PMV9	*Avulavirus*	217068695
*Avian Paramyxovirus 10*	Avian PMV10	*Avulavirus*	300432147
*Beilong virus*	Beilong	*Henipavirus*-like	Q287X8
*Bovine parainfluenza virus 3*	bPIV3	*Respirovirus*	P06162
*Canine distemper virus*	Canine DV	*Morbillivirus*	Q9DGW6
*Dolphin Morbillivirus*	Dolphin MV	*Morbillivirus*	1586312
*Bat paramyxovirus/Eid_hel/GH-M74a/GHA/2009*	Bat PMV	*Henipavirus*	Personal communication
*Fer de lance virus*	Fer de lance	*Ferlavirus*	34391488
*Hendra virus*	Hendra	*Henipavirus*	O55777
*Human parainfluenza virus 1*	hPIV1	*Respirovirus*	P32530
*Human parainfluenza virus*	hPIV2	*Rubulavirus*	P19847
*Human parainfluenza virus 2*	hPIV3	*Respirovirus*	P06163
*Human parainfluenza virus 4*	hPIV4	*Rubulavirus*	P21739
*J virus*	J virus	*Henipavirus*-like	Q49HN9
*Mapuera virus*	Mapuera	*Rubulavirus*	A3R041
*Measles virus*	Measles	*Morbillivirus*	Q9EMA9
*Menangle virus*	Menangle	*Rubulavirus*	82712718
*Mossman virus*	Mossman	*Morbillivirus*-like	Q6WGM4
*Mumps virus*	Mumps	*Rubulavirus*	P30927
*Nariva virus*	Nariva	*Morbillivirus*-like	B8XH60
*Newcastle disease virus*	Newcastle	*Avulavirus*	P0C765
*Nipah virus*	Nipah	*Henipavirus*	Q997F2
*Pacific salmon paramyxovirus*	Pacific PMV	*Respirovirus*-like	JF795583
*Parainfluenza virus 5*	PIV5	*Rubulavirus*	P11207
*Peste des petits ruminants virus*	PPRV	*Morbillivirus*	C3W4R0
*Phocine distemper virus*	Phocine DV	*Morbillivirus*	P35941
*Pigeon Paramyxovirus 1 (strain of Newcastle disease virus)*	Pigeon PMV1	*Avulavirus*	258547241
*Porcine rubulavirus*	Porcine RV	*Rubulavirus*	151266279
*Rinderpest virus*	Rinderpest	*Morbillivirus*	P60169
*Salem virus*	Salem	*Morbillivirus*-like	Q9IZC0
*Sendai virus*	Sendai	*Respirovirus*	P04859
*Simian virus 41*	SV41	*Rubulavirus*	P36315
*Tioman virus*	Tioman	*Rubulavirus*	Q91NG9
*Tuhoko virus 1*	Tuhoko1	*Rubulavirus*	298388482
*Tuhoko virus 2*	Tuhoko2	*Rubulavirus*	298388490
*Tuhoko virus 3*	Tuhoko3	*Rubulavirus*	298388498
*Tupaia paramyxovirus*	Tupaia PMV	*Morbillivirus*-like	Q9QM81

**Table 2:** Sequences of *Pneumovirinae*, *Filoviridae*, and *Rhabdoviridae* P protein.

Family or subfamily	*Genus*	Virus species	Accession number
*Pneumovirinae*	*Metapneumovirus*	Avian metapneumovirus	50898284
		Human metapneumovirus	46852134
	*Pneumovirus*	Human respiratory syncytial virus	9629202
		Bovine respiratory syncytial virus	9631271
		Pneumonia virus of mice	56900718
*Filoviridae*	*Ebolavirus*	Ivory Coast ebolavirus	B8XCN7
		Bundibugyo ebolavirus	B8XCM8
		Zaire ebolavirus	Q6V1Q9
		Sudan ebolavirus	Q5XX07
		Reston ebolavirus	Q91DE0
	*Cuevavirus*	LLoviu virus	353745024
	*Marburgvirus*	Marburg virus	Q1PD52
*Rhabdoviridae*	*Lyssavirus*	Rabies virus	P22363
		Australian bat lyssavirus	Q9QSP3
		European bat lyssavirus 1	A4UHP9
		Shimoni bat virus	D4NRJ9
		Mokola	P0C569
		West Caucasian bat lyssavirus	Q5VKP1
	*Vesiculovirus*	Vesicular stomatitis virus (VSV) Indiana	Q5VKP1
		Maraba virus	298563846
		Cocal virus	B3FRK7
		Vesicular stomatitis virus (VSV) Alagoas	B3FRL2
		Carajas virus	298563847
		Vesicular stomatitis virus (VSV) New Jersey	P04877
		Isfahan virus	Q5K2K6
		Chandipura virus	P16380
		Piry virus	Q01769
		Pike fry virus	C3VM12
		Spring viremia of carp virus	Q91DS2

### Sequence alignment and comparison

We generated multiple sequence alignments (MSAs) of the N-terminal moieties of the P of each *Paramyxovirinae* genus by using MAFFT [21] (version 6 with options L-INS-i). We also used the metapredictor M-coffee [22], ran with all default MSA programs with the exception of MAFFT: PCMA (version 2.0) [23], POA [24], DIALIGN-TX [25], Muscle [26], ProbCons [27], ClustalW [28] and T-Coffee [29]. We examined the reliability of the alignments using Guidance [30] (using the MAFFT option) and CORE [22] (which is part of the standard output of M-coffee [22]). These methods are complementary, since they rely on independent approaches (respectively robustness to changes in phylogenetic guide trees, and degree of agreement between several multiple alignment algorithms). We discarded parts of the MSAs that we did not consider to be reliably aligned.

**Figure 2:**
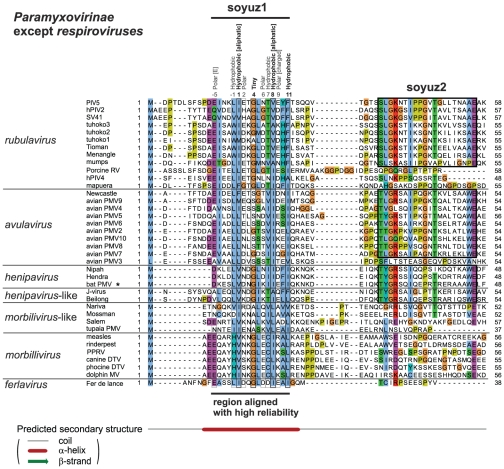
Alignment of the N-termini of P from all *Paramyxovirinae* except *respiroviruses* (see **Figure 3**), realized with MAFFT and coloured according to the ClustalX scheme [**33**]. Abbreviations and accession numbers are in Table 1. Positions with conserved physico-chemical character are indicated above the alignment, in bold if the character is strictly conserved (100%) and in normal font if it is generally conserved (>80%). Numbering of the soyuz1 motif (above the alignment) starts at the first strictly conserved position. Unpublished sequences are shown by an asterisk.

We compared in a pairwise fashion the MSAs of P of each *Paramyxovirinae* genus by making profile-profile comparisons with HHalign [31]. The threshold for statistically significant similarity was set at the commonly used value E = 1×10^−3^, and we also examined subsignificant similarities that had E-values between 1×10^−1^ and 1×10^−3^. To generate an MSA of the N-termini of all *Paramyxovirinae* P and examine its reliability, we proceeded as above. All alignments presented in the Figures were visualized using Jalview [32], with the ClustalX colouring scheme (see Figure 2b and 2d in [33]), and are available on request.

We followed the same approach for the P of other *Mononegavirales* families.

### Sequence motif discovery

We used the following programs (all ran from their web interface using default parameters) in order to identify over-represented sequence motifs in the N-termini of *Paramyxovirinae* P: MEME [34] (version 4.7.0), DILIMOT [35], and SlimFinder [36] (version 4.1).

### Nucleotide sequence analyses

The nucleotide alignments corresponding to the amino acid alignments of the N-termini of P were obtained using Protogene [37], which is part of the T-coffee suite at http://www.igs.cnrs-mrs.fr/Tcoffee/tcoffee_cgi/index.cgi. We used the metaserver WAR [38] to predict the secondary structure of RNAs.

In order to detect nucleotide constraints imposed by a potential RNA structure underlying soyuz1 or soyuz2, we examined visually the nucleotide variability at each codon position of the alignment. A constraint exerted mostly at the protein level would result in the second codon positions being the most conserved, and the third codon positions the least conserved. Conversely, departure from this pattern would indicate the presence of selection exerted at the nucleotide level.

### Protein sequence analyses

Secondary structure was predicted using Jpred [39]. Disordered regions were predicted using Medor [40], according to the principles described in [41]. We used Composition Profiler [42] to analyze the compositional bias (enrichment or depletion) of different regions in specific amino acids when compared to SwissProt (release 51).

The physico-chemical characters of amino acids are as follows (see also Figure 2d in [33]): aliphatic (IVL); hydrophobic (WFYMLIVACTH); alcohol (ST); polar (DEHKNQRST); tiny (AGCS); small (AGCSVNDTP); bulky (EFIKLMQRWY); positively charged, i.e. basic (KRH); negatively charged, i.e. acidic (DE); or charged (DEKRH).

To investigate the 3D structure of soyuz1 and soyuz2, we examined the three structures available for PIV5 V: a monomer of V bound to DDB1 alone (PDB accession number 2b5l, chains C and D) [43], and a monomer of V bound to the complex DDB1-CUL4-ROC1 (accession number 2hye, chain B), which is the one presented in Figure 7 [44]. Structural comparison between *Mononegavirales* P was carried out using FATCAT [45].

## Results

### The N-terminal tip of all *Paramyxovirinae* P, except *respiroviruses*, contain a common motif of 16aa, soyuz1

The N-termini of *Paramyxovirinae* P are globally alignable within each genus, but not between different genera. Therefore, we first generated multiple sequence alignments (MSAs) of the N-terminal moieties of the P of each *Paramyxovirinae* genus and then compared the MSAs in a pairwise fashion (see Material and Methods). HHalign reported statistically significant similarities between the first 50–60aa of *rubulavirus*, *avulavirus* and *henipavirus* P, with E-values around 1×10^−6^. This corresponds to the conserved region described previously in these genera only (described in Figure 7 of [5]). However, HHalign also reported subsignificant similarities (E>1×10^−3^) between the first 40aa of the P of other genera, for instance between *henipavirus* and *morbillivirus* P (E = 1.7×10^−3^) corresponding respectively to aa 7–26 of *Nipah virus* P and to aa 9–28 of *measles virus*, or between *henipavirus* and *respirovirus* P (E = 1.5×10^−3^), corresponding to aa 6–18 of *Nipah virus* P and aa 25–36 of *Sendai virus* P. Thus, the P of most *Paramyxovirinae* have a short region of marginal sequence similarity in their extreme N-terminus.

To investigate further this similarity, we aligned the first 60aa of *Paramyxovirinae* P using MSA algorithms classified among the best-performing in recent benchmarks, and examining their reliability using two complementary methods (see Material and Methods). A region of 16aa, which we called soyuz1, was reliably aligned in the N-termini of the P of all *Paramyxovirinae* except *respiroviruses* (Figure 2). Soyuz1 contains four positions with strict physico-chemical conservation (see Material and Methods for the classification of amino acids employed here). They are located in positions 1, 4, 8 and 11, shown in bold above the alignment in Figure 2 (numbering starts at the first position with strict conservation). Soyuz1 also contains 6 positions with good (>80%), but not strict, physico-chemical conservation, shown above the alignment in Figure 2. In all genera, soyuz1 was predicted to form a short α-helix, upstream of a long region devoid of secondary structure.

### Soyuz1 is also present in respirovirus P but in a shorter form of 11aa

**Figure 3:**
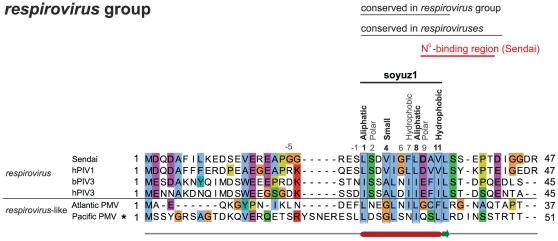
Alignment of the N-termini of P from *respiroviruses*. Positions matching the soyuz1 of the other *Paramyxovirinae* are indicated above the alignment (see Figure 2). An experimentally characterized substitution in *Sendai virus* is in bold.

**Figure 4:**
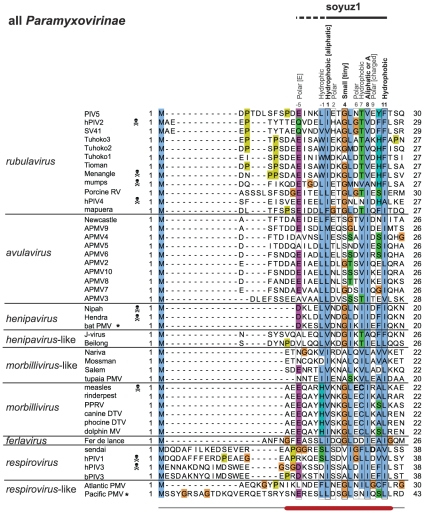
Alignment of the N-termini of P from all *Paramyxovirinae*. Conventions as in Figure 2. The part of soyuz1 not conserved in *respiroviruses* is indicated by a dashed line above the alignment. Species pathogenic for humans are marked by a skull and crossbones. Experimentally characterized substitutions in *measles virus* and *Sendai virus* are in bold.

We examined the N-terminus of P in the remaining genus, *respirovirus*. It is highly variable but we identified a short region (aa 25–36 in *Sendai virus*) predicted to form an α-helix, conserved in all *respiroviruses* and also in the related *Atlantic salmon paramyxovirus* (Figure 3). This region contains the same four conserved positions as soyuz1, if one allows in position 4 small aa, such as V (found in hPIV1 and *Sendai virus*), instead of only tiny aa (Figure 3). We aligned the first 60aa of all *Paramyxovirinae* P, including *respiroviruses*. MAFFT and M-coffee aligned the conserved region of *respirovirus* P with the soyuz1 of other *Paramyxovirinae* (see Figure 4), but the alignment was deemed less reliable by CORE and GUIDANCE. All generally conserved positions of soyuz1 were also conserved in *respiroviruses*, with the exception of positions −5 and −1. We conclude that *respirovirus* P also have a soyuz1 motif, albeit in a shorter version (11aa), starting at aa 1 instead of aa −5.

### Newly sequenced *Paramyxovirinae* P also contain a soyuz1 motif

We obtained two unpublished sequences of P: that of *bat paramyxovirus* (a new *henipavirus* isolated from African bats and kindly contributed by F.J. Drexler) and that of *Pacific salmon paramyxovirus* [46], [47] (related to *respiroviruses* and kindly contributed by J. Winton and B. Batts). We found both to contain the soyuz1 motif (Figure 4). In addition, while this manuscript was in preparation, the sequence of a new *Paramyxovirinae*, *Tailam virus*, related to *Beilong virus*, was published [48], and it also contains the soyuz1 motif (not shown).

In summary, in all *Paramyxovirinae*, i.e. 45 species including nine human pathogens (marked by a skull and crossbones symbol in Figure 4), P contains in its first 40aa a short motif, soyuz1, with predicted α-helical potential. Note that the protein V also contains the soyuz1 motif, since it has the same N-terminus as P (Figure 1).

### Soyuz2, a motif downstream of soyuz1 conserved in most *rubulaviruses*, *avulaviruses* and *henipaviruses*

**Figure 5:**
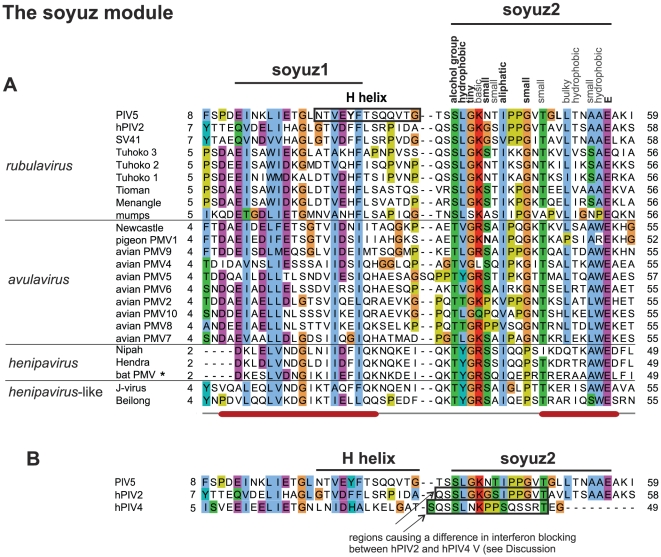
N-termini of P from the *rubulaviruses*, *avulaviruses*, and *henipaviruses* that have the soyuz2 motif. Conventions as in Figure 2. (A) Experimentally characterized substitutions in soyuz2 and in the H helix are in bold. (B) Comparison of the N-termini of the V protein of PIV5 and hPIV2 (which both have a soyuz2 motif) with that of hPIV4 (which lacks the soyuz2 motif).

**Figure 6:**
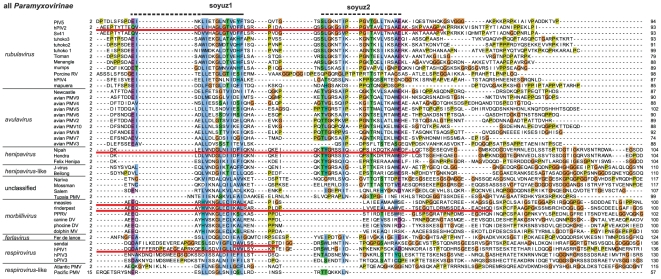
Alignment of the first 100aa of all *Paramyxovirinae* P. Conventions as in **Figure 2**. The boundaries of N°-binding regions (underlined in red) have generally been determined indirectly (Table 3), and thus should be taken as approximate. Regions downstream of soyuz1 and soyuz2 (90–330aa in length, of which only ∼50aa are visible on the figure) are unalignable between different genera of *Paramyxovirinae*.

A region of 20aa is conserved downstream of soyuz1 in *rubulaviruses*, *avulaviruses* and *henipaviruses*, with the exception of *hPIV4*, *mapuera virus*, *porcine RV* and *avian PMV3* (see Figure 2). We called this motif soyuz2 and present it in more detail in Figure 5. Its most striking feature is a strictly conserved E in last position. Soyuz2 corresponds to the second half of the conserved region we had previously detected (described in Figure 7 of [5]). However, the alignment of soyuz2 was incorrect because it mistakenly incorporated *hPIV4* and *porcine RV*, and as a consequence the alignment failed to reveal several conserved positions reported herein, including the strict conservation of E. We could find no region similar to soyuz2 in other viruses, with the exception of *Nariva virus* and *Mossman virus* (phylogenetically close to *morbilliviruses* and *henipaviruses*), which might have a degenerate version of the motif (Figure 2). The rest of P is extremely variable among *Paramyxovirinae* P (see Figure 6).

In summary, all *Paramyxovirinae* P contain a short motif, soyuz1, while some *rubulaviruses*, *avulaviruses*, and *henipaviruses* contain another motif, soyuz2, downstream of soyuz1. In these genera, soyuz1 and soyuz2 correspond respectively to the first and second half of the conserved region we had previously described [5]. However, the P of the three other *Paramyxovirinae* genera also contain a soyuz1 motif, previously undetected. In our previous work, we could detect soyuz1 using standard approaches such as psi-blast only because in some genera it occurs together with soyuz2, which is very well conserved. We could identify the presence of soyuz1 in the three remaining *Paramyxovirinae* genera only by carefully examining subsignificant similarities in profile-profile comparisons (in the present work).

### Soyuz1 is enriched in order-promoting and acidic residues, while soyuz2 is enriched in flexible and basic residues

We studied the amino acid composition of soyuz1 and soyuz2 (see Material and Methods). Globally, soyuz1 is significantly (P<0.01) depleted in the positively charged residue R and enriched in negatively charged (acidic) residues D and E. Soyuz1 is thus negatively charged or neutral in most species, with the exception of *morbilliviruses* and some unclassified species, which can be positively charged. Remarkably, soyuz1 never contains any Proline; this depletion is highly significant (P = 10^−6^). Given that Proline is strongly disfavored in helices, and that soyuz1 is consistently predicted as α-helical, this suggests that soyuz1 might need to form an α-helix to perform its function(s). Finally, soyuz1 is globally enriched in order-promoting, bulky, and hydrophobic aa (I in particular).

On the contrary, the soyuz2 motif is depleted in acidic residues (D in particular) and thus almost always positively charged. It is depleted in order-promoting residues and enriched in disorder-promoting ones.

In conclusion, soyuz1 is often negatively charged, is hydrophobic, and has a strong propensity towards α-helices, whereas soyuz2 is positively charged and likely to be highly flexible.

### Soyuz1 and soyuz2 are mostly in extended conformation in the only 3D structure available

**Figure 7:**
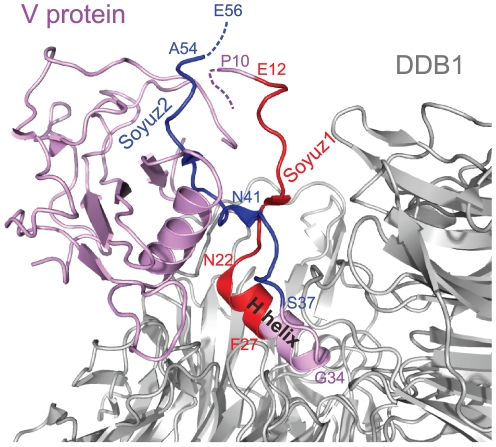
Structure of the V protein from *parainfluenza virus 5* bound to DDB1. The PDB accession number of the structure is 2HYE. Aa 1–9 and aa 55–80 of V, encompassing the last 2aa of soyuz2, are not visible in the crystal structure, presumably because they are disordered (see text). Soyuz1 is coloured red and soyuz2 blue. The H helix of V, bound to DDB1, is indicated; it partially overlaps with soyuz1.

As mentioned in the Introduction, the N-terminus of P has been found experimentally to be mostly disordered in many *Paramyxovirinae* (by disorder we mean lack of stable tertiary structure; this does not exclude transient secondary structure). However, the N-terminus of P has recently been observed in an ordered state, in the V protein of *parainfluenza virus 5* (PIV5), a *rubulavirus*, bound to the cellular protein DDB1 [43], [44]. In the structure, solved by X-ray crystallography, regions upstream of soyuz1 (aa1–9) and downstream of soyuz2 (aa 55–80) are not observable, presumably because they are disordered (they are indicated by dotted lines in Figure 7). In particular, the strictly conserved E of soyuz2 (E56 in PIV5) is not observable, which suggests that DDB1 is not the natural target of soyuz2.

Figure 7 represents the complex between DDB1 (in grey) and V (in purple), with soyuz1 in red and soyuz2 in blue. V is composed of two structurally independent elements [43], [44]: a non-globular moiety (aa 1–40, to the right-hand side of V in Figure 7), and a globular moiety (aa 41–222), to the left hand-side of V in Figure 7). The first moiety of V contains an α-helix, called the H helix (indicated by text in Figure 7), which provides the main contribution to binding DDB1, by inserting itself into a pocket of DDB1 [49]. The second moiety contains a seven-stranded β-sheet followed by a zinc finger. Only the first four β-strands are visible in Figure 7.

As can be seen in Figure 7, soyuz1 and soyuz2 mostly adopt an extended conformation with little regular secondary structure when bound to DDB1, with two exceptions: six aa of soyuz1 contribute to the beginning of the H helix (see also Figure 5), and two aa of soyuz2 contribute to the β-ladder, forming its first β-strand. Unfortunately, to our knowledge there is no experimental information regarding the structural state of soyuz1 or soyuz2 when not bound to DDB1.

### The conservation of soyuz1 or soyuz2 is not due to an underlying RNA structure

The conservation of soyuz1 and soyuz2 (see Figure 6) suggests a strong constraint. In theory, this constraint could result from the presence of an overlapping reading frame or an underlying RNA structure, rather than from selection acting at the protein level. Many *Paramyxovirinae (rubulaviruses*, *avulaviruses, ferlaviruses*) do not have a C reading frame that overlaps P [50], [51]; we therefore examined whether there was an overlooked RNA structure underlying soyuz1. We could not detect any predicted RNA structure (see Material and Methods). A simple analysis (not shown) of the nucleotide variability at each codon position of the alignment revealed no striking departure from constraints imposed by selection acting at the protein level (see Material and Methods). We conclude that an RNA structure cannot be the main reason for conservation of soyuz1, although we cannot exclude the presence of an RNA secondary structure forming non-canonical base pairs and undetectable by current programs [52], which might exert a weak constraint on the protein-coding sequence.

We performed the same analyses on soyuz2 (not shown), and again could detect neither a predicted RNA structure nor departure from sequence constraints operating at the protein level. Therefore, the conservation of soyuz2 most probably comes from a constraint at the protein level.

### The N°-binding site of *Paramyxovirinae* P encompasses soyuz1 or overlaps with it

The conservation of soyuz1 within an otherwise hypervariable region (see Figure 6), its hydrophobicity [53] and helical propensity are reminiscent of protein-binding regions that are disordered in isolation but can fold upon binding their target [54]. We searched the literature for functional information associated with soyuz1 and found that it is located within the N°-binding site of P in almost all *Paramyxovirinae* for which experimental data are available (Table 2 and Figure 6). This strongly suggests that soyuz1 plays a role in binding N°. The only exception is *Sendai virus*, a *respirovirus*, in which soyuz1 is not entirely encompassed within the N°-binding site of P but rather overlaps it by 3aa (see Table 3, Figure 3 and Figure 6). However, in the article that determined this N°-binding site [55], we noticed that the sequence reported as that of hPIV1 P was actually that of hPIV1 C. While this does not impact on the authors' experimental conclusions, it means that the region actually conserved in *respirovirus* P (aa 25–42 of *Sendai virus* P) is larger than that reported in their article (aa 32–42), and in fact encompasses soyuz1 (Figure 3).

**Table 3:** Functional information associated with the soyuz1 motif.

Genus	Species	Protein characterized^1^	Location of soyuz1 (aa)	Region characterized^2^	Function of that region^3^	References
*Rubulavirus*	PIV5	V	12–27	22–34 ( = H helix)	Binds to DDB1	[49]
*Rubulavirus*	hPIV2	P and V	11–26	1–46	Nuclear Localisation Signal	[109]
*Rubulavirus*	hPIV2	P and V	11–26	1–46	Binds to N°	[110], [111], [112]
*Henipavirus*	Nipah	V	2–17	1–50	Binds to N	[113]
*Morbillivirus*	Measles	P and V	4–19	1–20	Binds to N°	[56], [114]
*Morbillivirus*	Rinderpest	P	4–19	1–59	Bind to N	[93]
*Respirovirus*	Sendai	P	25–35	33–41	Binds to N°	[55]
*Respirovirus*	hPIV3	P	25–35	1–40	Binds to N°	[115]

1 P and V share the same N-terminus, containing soyuz1 (see Figure 1). We indicate whether the study was carried out on P or/and V.

2 The location of these functional regions have generally been determined indirectly, and should thus be taken as approximate boundaries.

3 In cases where the form of N was not characterized (either N° or the nucleocapsid), we report “N”.

Examining the effect of substitutions introduced into soyuz1 might yield further clues to its function(s). We could find only two studies that performed such substitutions. A double substitution (E14A - C15A) in *measles virus* V (in bold in Figure 4) caused only a very minor reduction in binding to N° [56], and the substitution D33G in *Sendai virus* P (in bold in Figure 3 and Figure 4) had no apparent effect on viral replication [55]. We note, however, that the effect of the former substitution was tested on V rather than P, and that these substitutions did not affect the four positions of soyuz1 that are strictly conserved physico-chemically (Figure 4).

### The N-terminal tips of other *Mononegavirales* P also contain conserved motifs

**Figure 8:**
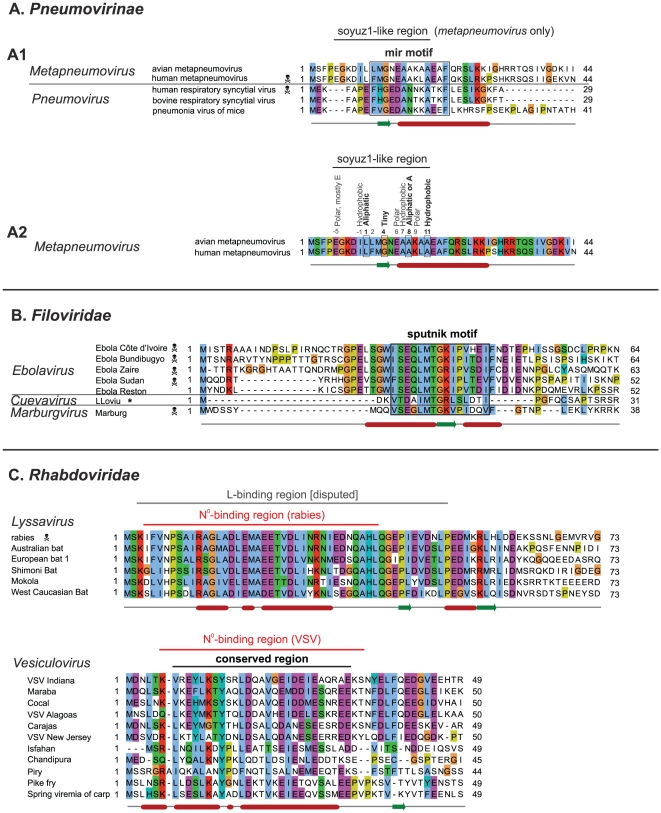
Alignments of the N-termini of P from *Pneumovirinae*, *Filoviridae* and *Rhabdoviridae*. Conventions as in Figure 2. Abbreviations and accession numbers are in Table 2. (A) Mir motif of *Pneumovirinae*. A1 – Alignment of the N-terminus of P of both *metapneumoviruses* and *pneumoviruses*. A2 – Same alignment as in A1 but restricted to *metapneumoviruses*. Positions corresponding to soyuz1 are indicated above the alignment. The coloring of sequence conservation is different from A1 since conservation is now based only on the two *metapneumovirus* sequences. (B) Sputnik motif of *Filoviridae*. The asterisk indicates the newly published sequence of *LLoviu virus*. (C) N-termini of the P of two *Rhabdoviridae* genera: *lyssavirus* and *vesiculovirus*. A disputed L-binding site in *lyssavirus* P is indicated [108]. The boundaries of the N°-binding region of VSV P were obtained from the crystal structure of N°-P [58].

Other *Mononegavirales* P have an organization similar to that of *Paramyxovirinae*, shown in Figure 1. We found that the P of most *Mononegavirales* have an N-terminal “tip” with features similar to those of soyuz1, i.e. a low variability and one or two predicted secondary structure elements located upstream of a variable region devoid of predicted secondary structure. In particular, all *Pneumovirinae* P have a conserved N-terminal motif, which we called mir (Figure 8A). Likewise, the P of all *Filoviridae* have a conserved N-terminal motif (Figure 8B), which we called sputnik (we could not find previous descriptions of these motifs in the literature). The similarity between the mir motif of *metapneumovirus* and *pneumovirus* P was not significant (E = 1.4×10^−3^), while the similarity between the sputnik motif of *ebolaviruses* and *Marburg virus* was significant (E = 1.4×10^−7^). Interestingly, while this manuscript was in preparation, the sequence of a new *Filoviridae*, *LLoviu virus*, was published [57], and it also contains the sputnik motif (Figure 8B).

We could find a conserved N-terminal region only in the P of three genera of the *Rhabdoviridae*: *vesiculoviruses*, *lyssaviruses* (Figure 8C), and *ephemeroviruses* (not shown), and there was no detectable sequence similarity between the genera. This might be related to the much higher overall sequence variability of *Rhabdoviridae* P when compared to other *Mononegavirales*. The N-terminal motifs of *Pneumovirinae* (Figure 8A) and *Rhabdoviridae* (Figure 8C) are predicted or known [58] to be α-helical, like soyuz1. The sputnik motif of *Filoviridae* is clearly different, since it contains a short predicted β-strand and a Proline (Figure 8B).

These N-terminal motifs have no detectable sequence similarity, with one potential exception. The mir motif of *metapneumoviruses* has striking similarity to soyuz1, matching 9 out of its 10 conserved positions (Figure 8, panel A1). Nevertheless, this similarity should be taken with caution since it is based on only two sequences, and since the mir motif of the other *Pneumovirinae* genus, *pneumovirus*, matches only two of the four characteristic positions of soyuz1, positions 4 and 11, and contains a Proline, absent from soyuz1 (Figure 8, panel A2).

The functions of the mir and sputnik motifs are unknown, to our knowledge, whereas the conserved N-termini of *Rhabdoviridae* P are known to bind N° (Figure 8C), like in *Paramyxovirinae* [59], [60]. The N°-binding region of VSV P has recently been determined precisely by X-ray crystallography [58], and it corresponds well to the region conserved in other *vesiculoviruses* (Figure 8C).

### The C-termini of Mononegavirales P contain a structurally similar region

**Figure 9:**
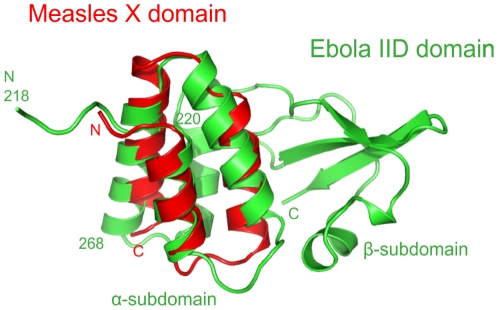
Structural superposition of the C-termini of two *Paramyxovirinae* and *Filoviridae* P. FATCAT superposition between the measles virus X domain (PDB accession number 1T60, chain A), in red, and the *Zaire ebolavirus* IID domain (3FKE, chain A), in green. N and C refer to N- and C-termini.

The common organization of *Mononegavirales* P and their common genomic location suggests that they may have originated from a common ancestor and we therefore looked in detail at potential structural similarities. Their multimerization domains are structurally dissimilar [61], [62], [63]. On first inspection, their C-terminal domains are also very different: they form a triple α-helix bundle in *Paramyxovirinae* (“X domain”) [64], [65], [66], a mixed α-β fold in *Rhabdoviridae* [67], [68], and an α-helix subdomain packed against a β-sheet subdomain in *Filoviridae* (Interferon Inhibitory Domain, IID) [69]. Nevertheless, we performed a similarity search on the recently solved structure of *Zaire ebolavirus* IID. FATCAT [45] reported the X domain of *Paramyxovirinae* P within the first 15 hits, superposing it well (P = 1.28×10^−3^, RMSD = 2.6 over 51 aa) with the first three helices of the α-helical subdomain of IID (aa 218–268, composing 39% of its residues) (Figure 9). We found that the C-terminal domain of the P of *rabies virus*, a *Rhabdoviridae*, also had weak structural similarity with the X domain of *measles virus* P (superposition over two α-helices only; not shown), as previously reported [70].

## Discussion

### The motifs we detected probably evolved by homologous descent

**Figure 10:**
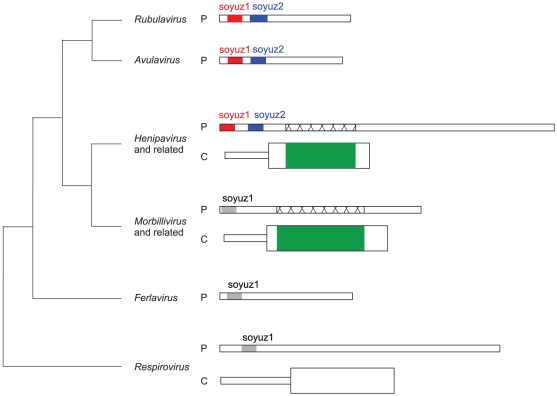
Regions with sequence similarity in *Paramyxovirinae* P and C. The N-termini of *Paramyxovirinae* P and the C proteins that overlap them are represented to scale (the N-terminus of *henipavirus* P is about 380aa long). The phylogenetic relationships between different genera are shown on the left as a cladogram based on [71]. Regions with statistically significant similarity (and thus homologous) are shown in the same colours, whereas regions that have subsignificant similarity are shown in grey. The crisscrossed regions of *henipavirus* and *morbillivirus* P are homologous, even though they have no detectable similarity, since they overlap homologous regions of C, in green (see Discussion).

The motifs we have identified are certainly not spurious, since they are also present in two distantly related viruses whose sequence was released after our main analysis. The fact that the motifs are present in all species within their respective families (for instance, soyuz1 is present in all 45 *Paramyxovirinae*) strongly suggests that they are functionally important. In theory, they could have originated either by convergent evolution or by homologous descent. The sequence similarity between the motifs of different genera is generally not statistically significant (except for the *Filoviridae* sputnik motif) and cannot by itself discriminate between these two hypotheses. However, in the case of soyuz1, we believe three points argue compellingly in favour of homologous descent. 1) Soyuz1 is demonstrably homologous in *rubulaviruses*, *avulaviruses*, and *henipaviruses*, since in these it has statistically significant similarity. 2) In all genera, soyuz1 is found in exactly the same position, within the first 40aa of P. This common location is much less likely to have originated by convergent evolution. 3) A part of C that overlaps P downstream of soyuz1 (in green in Figure 10) has distant, but statistically significant similarity among *henipaviruses*, *morbilliviruses* and related viruses (not shown). Therefore, the corresponding region of P (crisscrossed in Figure 10) is also homologous in these viruses. Thus, it is not only the C-terminal moiety of P, but almost all of P downstream of soyuz1 that is demonstrably homologous in *henipaviruses* and *morbilliviruses*. This considerably increases the probability that the similarity among their soyuz1 results from homologous descent. Lastly, we note that the fact that *respiroviruses* have a somewhat divergent soyuz1 motif is coherent with *Paramyxovirinae* phylogeny (Figure 10), in which *respiroviruses* are basal [71].

Similarly, the mir motif always occurs in the same position in *Pneumovirinae* P, arguing (albeit less strongly) for homologous descent.

### Soyuz1 probably binds N°

It seems unlikely that the conservation of soyuz1 results from binding a cellular partner involved in antiviral defense, because even closely related viruses often use different proteins or different regions of a protein to bind the same antiviral protein [72], [73]. Thus, we think that soyuz1 probably binds a conserved viral or cellular partner(s) indispensable to viral replication. One of these partners is almost certainly N°, since soyuz1 is encompassed within the N°-binding site of P in all species for which biochemical data are available (Table 2 and Figure 6). Accordingly, in the *rubulavirus* PIV5, the binding of P to N° is mostly of a hydrophobic nature, since it is abolished by detergent but not by strong salts [74]. This is consistent with it occurring through soyuz1, which is very hydrophobic. Intriguingly, the *respirovirus* N°-binding site, which has been mapped precisely to a stretch of 8aa, does not correspond exactly to soyuz1 but rather overlaps its first 3aa (Figure 3) [55]. This suggests that the soyuz1 of *respiroviruses*, which is divergent in sequence, might function differently from that of other *Paramyxovirinae*. Alternatively, the conservation of soyuz1 might be explained by it binding not only N° but also a second protein whose binding site partially overlaps with that of N° but extends upstream. This would provide an attractive mechanism to explain the initiation of encapsidation of the viral genome: by binding to soyuz1, this protein would provoke the release of N°, which would then be free to bind to nascent RNA. A candidate for this role might be the polymerase, L.

### Soyuz2, a role in inducing the proteasomal degradation of STAT proteins in *rubulaviruses*?

Soyuz2 is found in only three genera, but in these it is much more conserved than soyuz1 (Figure 2). This suggests that soyuz2 might interact with a cellular partner rather than a viral one. Despite its striking conservation, its function is unknown. However, we think that an elegant comparison between the V of *rubulavirus* hPIV2, which has the soyuz2 motif, and of hPIV4, which does not have it (see Figure 2), suggests a role for soyuz2 in proteasomal degradation of STAT proteins [75]. Both hPIV2 V and hPIV4 V bind the DDB1-cullin4-STAT1-STAT2 complex [75]. However, unlike hPIV2 V, hPIV4 V is incapable of triggering subsequent proteasomal degradation of STAT1 or STAT2, a key step in blocking interferon signaling [2], [76]. Nishio *et al.* [75] replaced a region of hPIV2 V corresponding almost exactly to soyuz2 by the equivalent region of hPIV4 V (boxed in Figure 5B). The exchange abolished the ability of hPIV2 V to block interferon signaling, strongly suggesting that soyuz2 plays a role in it. A study on the *rubulavirus* PIV5 provides additional support: a single substitution of soyuz2, L50P (in bold in Figure 5), decreased the capacity of V to block interferon [77]. Interestingly, this decrease was enhanced by an additional substitution, Y26H, in the H helix that binds DDB1 (Figure 5). Thus, although the great majority of studies on V have focused on its conserved C-terminus [2], [76], soyuz2 should also be the subject of investigations. The V proteins of *henipaviruses* and *avulaviruses*, which also contain a soyuz2 motif, inhibit the action of STAT1 through mechanisms different from *rubulaviruses* [78], [79], [80]. Nevertheless, in view of the conservation of soyuz2, it is tempting to speculate that in the three genera the inhibition of STAT1 might rely on a common cellular target with which soyuz2 interacts. We note that a substitution mapped within soyuz2, N37D (in bold in Figure 5), enhanced replication and virulence of Pigeon paramoxyvirus 1, an *avulavirus* [81]. Further studies are needed to determine whether it caused an effect on interferon signaling or on replication, and whether P or V was involved.

### The P of *Mononegavirales* probably share a common origin

This study and another [70] have detected a structural similarity between two α-helices of the C-terminal domains of *Paramyxovirinae*, *Rhabdoviridae*, and *Filoviridae* P. Several arguments suggest that this similarity, although weak (subsignificant), might be the result of common ancestry: the P proteins are encoded by genes with the same location and have a similar organization; the similarity occurs between domains occupying the same position within P; and finally, the structurally similar regions have the same function: they bind the viral nucleocapsid [70], [82], [83]. A common origin of domains that have different structural folds might seem improbable, but other examples are known [84] and the two α-helices might correspond to “elementary functional loops”, which are conserved structural and functional elements proposed to form building blocks of ancestral proteins [85].

### A similar role for the N-termini of *Mononegavirales* P to that proposed in the *Paramyxovirinae*?

All *Mononegavirales* N can self-assemble illegitimately on cellular RNA [86], [87], [88], [89], with the exception of *Bornaviridae* [90], [91]. In both *Paramyxovirinae* and *Rhabdoviridae*, the N-terminus of P binds N° and keeps it unassembled [5], [55], [59], [60], [92], [93]. In view of their probable common origin (see above), it would be interesting to investigate whether in *Pneumovirinae* and *Filoviridae* it is also P that prevents the assembly of N°, and whether binding occurs through mir and sputnik. Interestingly, in *pneumonia virus of mice*, a *pneumovirus*, a region containing mir has been reported to bind N [94], though what form of N was bound was not studied. We found no published data regarding sputnik, but *Zaire ebolavirus* VP35 mutants lacking sputnik did not support viral replication or transcription, though they were still able to block interferon induction (Grosch and Mühlberger, personal communication).

### Our approach should allow the identification of previously overlooked short, disordered domains

It has been recently proposed that conserved, disordered regions longer than 20–30aa form a new type of binding elements: “disordered domains”, which fold into specific structures upon binding their target [95], [96], [97]. These regions often constitute functional, evolutionary and structural units (hence the name “domain”), and were thought to clearly differ from shorter elements, in particular linear motifs (3–11aa) [98], through their binding mode, affinity, and the fact that they arise by homologous descent rather than convergent evolution [95]. Reliable *in silico* identification of disordered domains would be a major advance because they mediate numerous (possibly thousands) of crucial but poorly characterized protein-protein interactions [99]. So far their detection has been restricted to domains longer than 20–30aa [95] because similarities detected between shorter regions are not statistically significant.

Our study shows that carefully examining disordered regions of orthologous proteins allows the detection of shorter regions, such as soyuz1 (11–16aa), which most probably evolved by homologous descent. We expect our approach to detect short disordered domains even in hypervariable, very long regions (up to 380aa for soyuz1). Further improvements in their detection could come from progress in aligning disordered regions [100], [101]. Our approach should also be applicable to prokaryotes and eukaryotes, whose orthologs are available in dedicated databases that greatly facilitate their collection [102].

An alternative approach to identify sequence motifs could rely on dedicated software such as MEME [34], DILIMOT [35], and SlimFinder [36]. Using these programs with default parameters (see Material and Methods), we were unable to fully recover all instances of soyuz1 and soyuz2. This could be due to the fact that the programs are optimized to detect shorter motifs (3–11aa), and are not intended to detect them within very long regions. Nevertheless, we think that these methods could be complementary for future research, especially since they have the advantage of being fully automated. Finally, we note that in principle our approach is also applicable to the discovery of motifs in ordered regions, though this was not the focus of this study.

### An approach to detect new drug targets?

In conclusion, experimental studies are now needed to identify the soyuz1-binding site on N°, elucidating what triggers the release of soyuz1 by N° during replication, and to identify the function(s) of soyuz2. The use by viral proteins of short peptides located within flexible regions to bind other viral proteins is emerging as a common pattern, found for instance in the interactions between PB1, PA and PB2 in *influenza virus* [103], [104], [105], and antiviral approaches aimed at disrupting these interactions are being tested [106]. The motifs found by our approach have the double advantage that they are plausible Achilles' heels of viruses (as suggested by their exceptional conservation) and are found in a wide range of human pathogens. If their biochemical role were confirmed, they might thus constitute new, attractive antiviral drug targets. Recently, Castel *et al.* [107] have provided a proof of concept for this idea by using a peptide mimicking the N°-binding site of P to inhibit the replication of *rabies virus*, a *Rhabdoviridae*.
